# Editorial: The psychology of parenting in unique life experiences: understanding the challenges of continuous stressful circumstances and marginalized populations

**DOI:** 10.3389/fpsyg.2025.1680236

**Published:** 2025-09-02

**Authors:** Tal Araten-Bergman, Ayelet Gur, Uri Yatzkar, Vered Shenaar-Golan

**Affiliations:** ^1^School of Allied Health, Human Services and Sports, and Living With Disability Research Centre College of Science, Health and Engineering, La Trobe University, Melbourne, VIC, Australia; ^2^Social Work Department, Faculty of Social Sciences and Humanities, Tel-Hai College, Qiryat Shemona, Israel; ^3^Research Center for Innovation in Social Work, Faculty of Social Sciences and Humanities, Tel-Hai College, Qiryat Shemona, Israel; ^4^Tel-Hai College and Ziv Hospital Joint Research Center for Mental Health, Zefat, Israel; ^5^Faculty of Medicine, Bar-Ilan University, Ramat Gan, Israel; ^6^Ziv Medical Center, Zefat, Israel

**Keywords:** parenting, stress, marginalization, emotion regulation, disability, wellbeing

## Introduction

Parenting is one of the most profound and enduring responsibilities in human life. It involves shaping and nurturing another human being while balancing personal capacities, social expectations, and changing life circumstances. Parenting affects not only parents' own wellbeing but also children's development and, ultimately, the resilience of society.

For many families, parenting unfolds under chronic stress and structural marginalization. These contexts—marked by uncertainty, limited resources, social exclusion, and complex care needs—demand sustained coping while facing systemic barriers.

This Research Topic, *The Psychology of Parenting in Unique Life Experiences*, brings together seven studies exploring the lived realities of parents in such conditions. Using varied methods and theoretical perspectives, these works illuminate the interplay of psychological, relational, and structural factors shaping parenting under sustained adversity.

## Psychological foundations of parenting under stress

Three studies focus on the inner worlds of parents facing continuous challenges.

Muñoz-Peña et al. examine parents with chronic pain, showing that parental stress undermines competence, which then increases guilt, leading to higher depression risk and reduced parental wellbeing.

Shpigelman and Karlinski Argi explore Israeli mothers with lifelong physical disabilities, finding that motherhood heightens tensions between dependence and independence, shaping both disability and motherhood identities.

Klein et al. study parents of children with externalizing behavior problems, revealing that low differentiation-of-self predicts more need-frustrating parenting, with fathers' need-supportive practices linked to fewer child symptoms.

Together, these studies show that emotional regulation, identity integration, and perceived competence are central to sustaining parenting capacity under ongoing stress.

## Adapting to extraordinary and changing contexts

Parenting is shaped by external crises and societal shifts. Shnitzer-Meirovich et al., studying Israeli parents of children with autism during wartime, find that children's behavioral problems and parents' emotion regulation difficulties each predict parental burnout, with emotion regulation mediating this link.

In China, Xu et al. show that marital satisfaction mediates the relationship between maternal stress and burnout, while higher socioeconomic status unexpectedly intensifies the negative impact of stress on marital satisfaction.

These findings illustrate that effective parenting in crisis relies on emotional flexibility, strong relationships, and the capacity to adapt rapidly to changing demands.

## Beyond the family: social worlds and structural pressures

Parenting is embedded within broader social structures. Naicker et al. examine parents' resolution to an autism diagnosis, showing that parental hope mediates the link between resolution and reduced stress. Hope here reflects confidence in supporting the child and being supported by others.

Adedeji et al. review the limited research on parents of children with achondroplasia, identifying gaps in measurement tools and highlighting the roles of medical complexity, stigma, and fragmented support systems. They stress that resilience depends on access to inclusive services, information, and societal acceptance.

Together, these works emphasize that resilience in marginalized contexts requires more than individual coping—it demands equitable policies, responsive services, and supportive social environments.

## Conclusion

The contributions in this Research Topic underscore that parenting under chronic stress is not only a personal challenge but also a societal issue. Parents' wellbeing depends on psychological resources, supportive relationships, and systemic scaffolding. External crises and structural inequities magnify these needs, making integrated interventions essential.

By linking emotion regulation, identity, and hope with the influence of social and structural contexts, this Research Topic offers actionable insights for researchers, practitioners, and policymakers. Future interdisciplinary work must bridge psychology, social policy, and lived experience to foster environments in which all families—especially those facing sustained adversity—can thrive.

